# Compositional versatility enables biologically inspired reverse micelles for study of protein–membrane interactions†

**DOI:** 10.1039/d5sm00033e

**Published:** 2025-04-04

**Authors:** Sara H. Walters, Rachel L. Signorelli, Allyson G. Payne, Alimohammad Hojjatian, Brian Fuglestad

**Affiliations:** a Department of Chemistry, Virginia Commonwealth University Richmond Virginia USA fuglestadb@vcu.edu; b Office of the Vice President for Research and Innovation, Virginia Commonwealth University Richmond VA 23298 USA; c The Center for Drug Discovery, Virginia Commonwealth University Richmond VA 23298 USA

## Abstract

The study of membranes and their associated proteins is critical for understanding cellular processes. *In vitro* investigations utilizing membrane models often have limitations in their biological relevance due to the dissimilarity of experimentally compatible membrane mimetics to biological membranes. Development of membrane models that better mimic cellular membranes enables more biologically accurate observations of membrane associated proteins. In this work, we present upper tolerance concentrations for a range of lipids incorporated into reverse micelles (RMs), confirmed with dynamic light scattering (DLS). A breadth of lipid incorporation enabled biologically inspired RMs to be formulated based on the molar ratios of lipids present in eukaryotic membrane leaflets. Three systems were formulated matching lipid compositions of the inner leaflet of the plasma membrane (PM-RM), the outer mitochondrial membrane leaflet (MI-RM), and the outer rough endoplasmic reticulum membrane leaflet (ER-RM). The biologically-inspired RM formulations were characterized using DLS and cryo-electron microscopy (cryo-EM) and were found to have favorable properties for protein encapsulation. All three biologically inspired RM formulations effectively encapsulated fatty acid binding protein 4 (FABP4), a protein which shuttles fatty acids between membranes, confirmed by NMR. Also presented in this work is the first known high-resolution observation of the membrane-bound state of sterol carrier protein 2 (SCP2), a protein responsible for transporting an array of lipids between membranes. SCP2 was successfully encapsulated within all three RM systems, enabling NMR observation of the membrane interface of SCP2. The tolerances and formulations reported here allow for tailoring of RMs to mimic specific cellular membranes and will enhance studies of protein interactions with lipids and membranes among other investigations.

## Abstract 2

Reverse micelle formulations reflecting the lipid compositions of cellular membranes are presented. These biologically inspired membrane models allow high-resolution mapping of protein–membrane interfaces in a physiologically relevant context.

## Graphical abstract

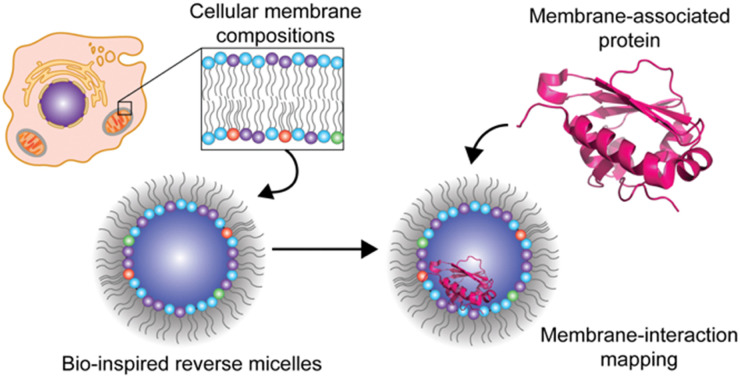

## Introduction

Cellular membranes and their associated proteins are the focus of intense study due to their fundamental roles in driving biological and disease related processes.^1^ Membrane structure and function is related to composition, with content of biological membranes varying through the identity of constituent lipids,^2^ their molar ratios,^3,4^ tail length and saturation,^5^ and other factors. The lipid composition of membranes differs between cell and tissue type,^5–8^ organism,^9,10^ organelle^11,12^ and are often asymmetrical, leading to differences among leaflets.^4^ Each lipid plays an important role within membranes, driving physical properties and biological function, and is therefore critical to the study of membranes and their associated proteins.^5^ Among lipids found in eukaryotic membranes, phosphatidylcholine (PC) is typically one of the main components and often comprises the majority of lipids.^2^ Other structural lipids include phosphatidylethanolamine (PE), phosphatidylserine (PS), phosphatidylinositol (PI), and phosphatidic acid (PA), many of which also modulate membrane-associated protein function among other roles.^2,5,13^ Other less abundant lipids are important for signaling, including lyso PC (LPC), lyso PA (LPA), some sphingolipids, phosphorylated PIs (PIPs), and cardiolipin (CL).^2^ The variety of lipids and distinctiveness of each cellular membrane presents a challenge in constructing biologically accurate models for *in vitro* studies.

Membrane associated proteins are housed in or interact with membranes, performing a variety of functions.^14,15^ Membrane models are necessary for studying integral membrane proteins or other membrane-adhered proteins *in vitro*.^16^ High-resolution study of membrane associated proteins has advanced recently, with cryo-EM being an effective tool for studying large, membrane associated proteins and complexes.^17^ However, a lower size limit excludes proteins such as small transmembrane proteins (TMPs)^18^ and single-domain peripheral membrane proteins (PMPs),^19^ which are water soluble but interact with membranes to perform function. Since crystallography is challenging for this type of protein, NMR is currently the best suited experimental method for many small membrane associated proteins.^1^ High-quality investigation of membrane associated proteins is dependent on the biological accuracy and experimental compatibility of available membrane models.^20^ Current membrane models typically consist of one or two surfactants or lipids, resulting in models that do not fully capture the complexity of membranes.^16,21,22^ Micelles are often comprised of non-natural detergents and are often destabilizing to proteins.^23,24^ Bicelles and nanodiscs are more bilayer-like models and may even be constructed from native membrane lipids.^23,24^ While these models have proven useful, they are large and often require extensive deuteration of the protein and/or lipids to collect high-quality NMR data, which increases material cost and may limit the information gained. Recent development of reverse micelles (RMs) as membrane mimetics has expanded the available tools for studying membrane associated proteins.^25^

RMs are a nanoscale pool of water surrounded by a lipid or surfactant shell solubilized in an apolar solvent such as an alkane.^26^ RMs have been used in NMR studies of aqueous proteins for inducing confinement,^27,28^ enhancing tumbling,^29,30^ studying surface hydration dynamics,^31,32^ and extending the detection limit in small-molecule binding for inhibitor design applications.^33,34^ The interior surface of RMs may be used to mimic membrane surfaces, allowing study of membrane properties and membrane associated proteins.^25^ Membrane mimicking reverse micelles (mmRMs) were recently formulated to imitate the interaction between PMPs and their biological membranes, while possessing favorable properties for protein NMR.^35^ This formulation has also been applied to fragment-screening for inhibitor design of membrane-adhered proteins.^36^ Native reverse micelles (nRMs) have been formulated from polar lipid extracts, including those from bovine heart, porcine brain, and soybean lipids.^37^ The tested nRMs contained a wide array of natural lipids and enabled the expected membrane interactions for membrane associated proteins. Although nRM systems contain a variety of natural lipids, the lipid extracts used to construct nRMs are from homogenized plant or animal tissues and do not reflect the relative lipid content of specific cellular membranes.

Here, we extend protein NMR compatible RM formulations towards the lipid content of specific cellular membranes and leaflets. We test tolerances of the mmRM system to incorporate a variety of lipids, formulate mmRMs to match specific cellular membrane lipid content, and demonstrate utility in protein NMR using two lipid transport proteins (LTPs); fatty acid binding protein 4 (FABP4) and sterol carrier protein 2 (SCP2). LTPs often weakly and transiently interact with membranes, carrying lipids through the aqueous compartments of cells.^38^ While their membrane interactions are often too weak for direct observation, confinement within RMs may favor the membrane bound state, as demonstrated previously with FABP4 in mmRMs and nRMs.^37^ We capture this interaction using mmRM formulations that correspond to the lipid content of specific cellular membranes. We further demonstrate utility by encapsulating SCP2, also called non-specific lipid transfer protein, a 13.3 kDa promiscuous lipid transporter.^39–45^ To our knowledge, SCP2 has only been studied at high-resolution in its aqueous state and results here are the first direct observation of membrane-bound SCP2. Successful encapsulation of membrane-bound LTPs here demonstrates high-resolution protein NMR in membrane models with more biologically accurate compositions. Biologically inspired mmRMs promise to provide a powerful tool for more accurate study of membrane associated proteins by NMR and other methods.

## Experimental

### Protein preparation

FABP4 and SCP2 were prepared using previously published protocols as a guide.^37,46^ All proteins were expressed with N-terminal poly-histidine affinity tags with TEV protease cleavage sites. Proteins were isotopically labeled with ^15^NH_4_Cl, and with ^13^C-d-glucose when needed, in *E. coli* using M9 minimal media. Protein expression was induced with isopropyl-β-d-1-thiogalactopyranoside (IPTG). Purification was performed using Ni-NTA affinity chromatography. N-Terminal polyhistidine tags were removed from the proteins using a TEV protease and the final concentrations were determined *via* Bradford Assay. FABP4 was found to bind to *E. coli* endogenous lipids and this form of the protein was used for encapsulation. SCP2 was delipidated using Lipidex-5000 resin, confirmed by NMR, and the apo-form was used for further encapsulation studies. Details of the protein preparations are included in the ESI.†

### Reverse micelle construction and tolerance screening

RMs were formed by adding an alkane solvent, either hexane or pentane, to an appropriate mass of surfactants and lipids which were prepared by drying overnight in a vacuum centrifuge. All lipids and surfactants were supplied by Avanti Polar Lipids and Echelon Biosciences. A total of either 75 mM or 100 mM total lipids and surfactants were used.^47^ Buffer volume corresponding to a defined water loading (*W*_0_) was then added to the solution. *W*_0_ is defined as the ratio of the concentration of water to the total concentration of surfactants and lipids in a RM sample and a *W*_0_ value of 20 was used in this study. 1-Hexanol is a necessary cosurfactant and was titrated following the addition of buffer, stopping when visual clarity was reached, usually around 1–1.2 M 1-hexanol. The RM sample was vortexed between each addition of 1-hexanol and briefly sonicated in a water bath if necessary. For protein encapsulation, lyophilized protein was added to the pre-constructed RM after visual clarity was reached by transferring the RM solution to a glass vial containing dried protein and vortex mixing, or were solubilized in buffer before adding. Samples were then left at room temperature on a shaker overnight and protein NMR data collection occurred the following day.

Tolerance screening of lipids was performed by adding the appropriate mass of the lipid of interest to DLPC:DPC mmRMs, followed by addition of the appropriate volume of aqueous buffer and titration of 1-hexanol. DLPC and DPC concentrations were lowered to accommodate additional lipids while maintaining a constant total lipid and surfactant concentration of 75 mM. The 1-hexanol phase diagram for each tested lipid was completed by titrating the RM to 2 M 1-hexanol, assessing visual clarity, and confirming stability, small size, and uniformity *via* DLS. Tolerance ranges were tested using a value above the maximum concentration of each lipid present in the cellular membranes that were selected for membrane-specific formulations. The membrane specific formulations were determined from reported values.^3,7,10,48–51^

### Dynamic light scattering

RMs used for lipid tolerance tests and membrane specific RM formulations were verified as small and monodisperse species using dynamic light scattering (DLS) size distributions, a hallmark of RM formation.^52,53^ All DLS experiments were performed on a Malvern Zetasizer Nano-S instrument. Viscosity and dielectric parameters were determined based on published literature for a binary system with hexanol and hexane.^54,55^ Measurements were collected at room temperature in a quartz cuvette. Each experiment was performed in triplicate with 12–18 scans per measurement with standard error calculated based on the triplicate run and the standard deviation based on the distribution of diameter values.

### Cryo-electron microscopy

The initial concentrations of all RM stock solutions were 100 mM, which were diluted 50 000-fold prior to use based on initial imaging screening results. Cryo-EM grids were prepared by applying 4 μL of the diluted solutions onto 300-mesh Quantifoil R2/2 copper grids (without glow-discharging) and plunge freezing them into liquid ethane cooled by liquid nitrogen using a Vitrobot Mark IV (set to 95% humidity, 4 °C, blot time: 3 seconds, blot force: 15 and wait time: 10 seconds). Multi-frame electron micrographs were collected on a Tundra electron microscope operated at 100 kV, equipped with a Ceta-F electron detector. Movies were recorded at a nominal magnification of 140 000× corresponding to a pixel size of 0.97 Å using the EPU software. All specimens were imaged with a total dose of 35 e Å^−2^. Preprocessing of the datasets (Motion correction and dose-weighting) were performed using CryoSPARC Live and the resulting micrographs were low-pass filtered at 6 Å to reduce noise.^56^

### NMR spectroscopy and analysis

Aqueous NMR samples consisted of ^15^N-isotopically labeled protein in its respective buffer at approximately 120–170 μM per sample, with 10% D_2_O added as a lock solvent. The FABP4 buffer consisted of 20 mM Tris pH 7.4, 100 mM NaCl, and 2 mM DTT. SCP2 buffer components included 20 mM Tris or Bis-Tris pH 8.5 or 6.0, 100 mM NaCl, and 2 mM DTT. For all RM samples, 10% d-pentane (Sigma-Aldrich) was added as the lock solvent. ^15^N-HSQC and SCP2 assignment experiments (HNCACB,^57^ CBCAcoNH,^58^ and HNCO^59^) were collected on 600 and 700 MHz Bruker AVANCE III instruments equipped with room temperature QXI probes. All experiments were conducted at 25 °C or 37 °C, as noted. NMR data was processed with NMRPipe and analyzed with the NMRFAM-Sparky distribution.^60,61^

## Results and discussion

**Fig. 1:**
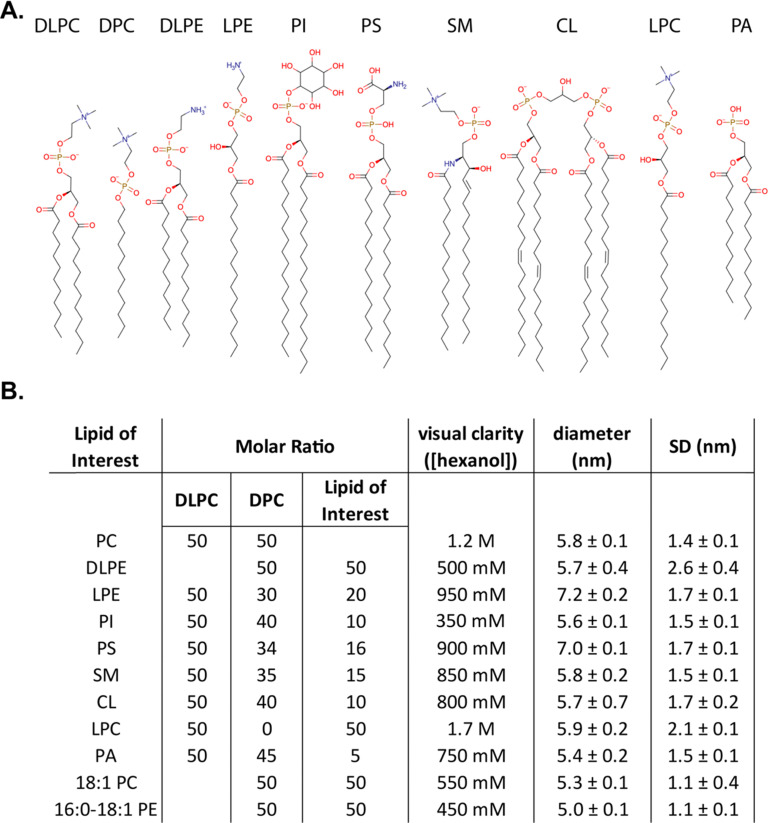
Surfactants used in this study and results of mmRM lipid tolerance testing. (A) Structures of the surfactants used to form bio-inspired reverse micelles. (B) Maximum lipid and surfactant concentrations used in each RM tolerance test, hexanol concentration needed for the formation of reverse micelles, and size and distribution parameters measured for each formulation from DLS. Sizes are reported as average diameters and their associated standard error from triplicate measurements. Standard deviation (SD) reflects the average width of the DLS distribution, reported with standard errors, from triplicate measurements.

To understand the ability of mmRMs to incorporate a breadth of lipid types, tolerances were tested for a series of lipids (Fig. 1(A)) incorporated into the established phosphocholine (PC) based 1,2-dilauroyl-*sn-glycero*-3-phosphocholine and *n*-dodecylphosphocholine (DLPC:DPC) mmRM system. For protein NMR applications, this system typically uses a 50 : 50 molar percent ratio of DLPC to DPC, with 75 – 150 mM total surfactant and typically 800–1200 mM hexanol as a cosurfactant in an alkane solvent such as pentane or hexane.^35,47^ The lipids all had a carbon tail length between 12 and 18 carbons to limit the size of the RMs, with a goal of reducing rotational correlation time for eventual use in protein NMR. All RMs used for tolerance testing were constructed with 75 mM total surfactants and lipids (Fig. 1(B)). Tested lipids represent major phospholipid classes found in eukaryotic membranes; 1,2-dilauroyl-*sn-glycero*-3-phosphoethanolamine (DLPE), 1-palmitoyl-2-hydroxy-*sn-glycero*-3-phosphoethanolamine (LPE), l-α-phosphatidylinositol (PI), 1,2-dipalmitol-*sn-glycero*-3-phosphoserine (PS), sphingomyelin (SM), 1′-3-bis[1,2-dioleoyl-*sn-glycero*-3-phospho]-glycerol (CL), 1-palmitoyl-2-hydroxy-*sn-glycero*-3-phosphocholine (LPC), and 1,2-dilauroyl-*sn-glycero*-3-phosphate (PA). Up to 37.5 mM was tested for DLPE and LPC, up to 15 mM for LPE, up to 12 mM for PS, up to 11.25 mM for SM, up to 7.5 mM for PI and CL, and up to 3.75 mM for PA with the remainder of the RM being DLPC and DPC. The upper-level tolerance values were chosen as a value higher than the molar percentage found in cytosolic facing eukaryotic membrane leaflets that were selected for eventual mmRM formulation: the plasma membrane inner leaflet,^3,48^ the outer mitochondrial membrane outer leaflet,^10,49,50^ and the rough endoplasmic reticulum outer leaflet.^7,50,51^ A water loading value (*W*_0_, molar ratio of water to surfactants and lipids) of 20 was used for all formulations of RMs, both with and without protein, based on the previously determined optimal W_0_ range for the DLPC:DPC formulation.^35^ Hexanol was not included upon initial construction and was titrated until visually clarity of the RM samples were achieved, which allowed construction of a hexanol-dependent phase diagram (ESI,† Fig. S1). All mmRM formulations formed visually transparent RMs with the addition of hexanol, often between 0.8 and 1.2 M. DLS was used to determine whether each lipid would integrate into the RM and allow formation of small and monodisperse RMs, which is important for protein NMR studies. All tested RM formulations were determined to be small and with a uniform distribution *via* DLS (ESI,† Fig. S2). Tolerance for two unsaturated lipids, 1,2-dioleoyl-*sn-glycero*-3-phosphocholine (18:1 PC) and 1-palmitoyl-2-oleoyl-*sn-glycero*-3-phosphoethanolamine (16:0-18:1 PE), were also tested to demonstrate the versatility of these RM models. Both unsaturated lipids formed RMs containing 37.5 mM of the lipids with 37.5 mM of DPC and maintained comparable size and uniformity expected from a RM (Fig. 1(B) and ESI,† Fig. S1, S2). All diameters measured using DLS were approximately between 5.0 nm to 7.2 nm, a size range indicating that small RMs are formed (Fig. 1(B)) and indicating a particle size that is amenable for use in protein NMR. By respecting the maximum concentrations tested here, and titrating to the appropriate amount of hexanol, any of the tested lipids, with a DLPC and DPC background, can be used to form RMs. This suggests flexibility in developing membrane models customized for the individual protein being studied and allows tuning of the system to include lipids of interest or to reflect specific biological membranes.

**Fig. 2:**
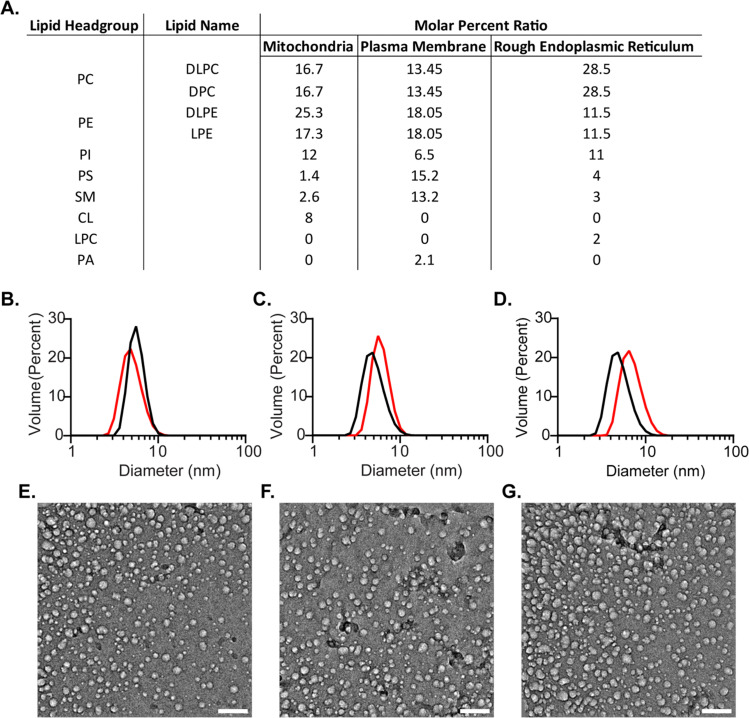
Biologically inspired membrane mimicking reverse micelle (mmRM) formulations and characterization by DLS and cryo-EM. (A) Molar ratios for each bio-inspired formulation constructed from 100 mM surfactants total. (B) DLS data for MI-RM with (black, diameter: 5.7 ± 0.1 nm, standard deviation: 1.2 ± 0.1 nm) and without FABP4 (red, diameter: 5.1 ± 0.1 nm, standard deviation: 1.4 ± 0.1 nm). (C) DLS data for PM-RM with (black, diameter: 5.6 ± 0.1 nm, standard deviation: 1.5 ± 0.1 nm) and without FABP4 (red, diameter: 6.1 ± 0.1 nm, standard deviation: 1.4 ± 0.1 nm). (D) DLS data for ER-RM with (black, diameter: 5.1 ± 0.1 nm, standard deviation: 1.5 ± 0.1 nm) and without FABP4 (red, diameter: 7.1 ± 0.1 nm, standard deviation: 2.0 ± 0.1 nm). Diameters reported here are fitted averages from three DLS measurements, standard deviations reflect the width of the distributions and are averaged among three DLS measurements. Standard errors are included for each. Cryo-EM images of E. MI-RM, F. PM-RM, and G. ER-RM confirm approximately spherical shapes. Scale bars are 50 nm.

To formulate more biologically accurate models, we sought to incorporate mixtures of lipid types into mmRM systems that reflect the assortment found in eukaryotic cellular membranes. Lipid types that are known to comprise over 1 molar percentage within the selected membranes were included. Three new mmRM formulations were derived from published lipid content from major cytosolic-facing membrane leaflets: the inner leaflet of the plasma membrane (PM-RM),^3,48^ the outer leaflet of the mitochondrial membrane (MI-RM),^10,49,50^ and the outer leaflet of the rough endoplasmic reticulum membrane (ER-RM).^7,50,51^ For all three formulations, the PC fraction of the lipid molar ratio was divided between DLPC and DPC, while the phosphatidylethanolamine (PE) fraction was divided between DLPE and LPE. While the LPE content deviates from the lyso-lipid content found in biological membranes and DPC is not a natural lipid, these were incorporated to retain a significant percentage of single-tail surfactants, which seems to be critical for forming mmRMs.^35,37^ We attempted to replace the DPC with LPC, but the RMs never fully formed, even with 2 M hexanol. The lipid compositions of each membrane-mimicking formulation are given in Fig. 2(A). The formulations for all three membrane models successfully formed visually transparent RMs with 100 mM total lipids. MI- and PM-RMs were successfully formed with 800 mM hexanol as the cosurfactant and the ER-RM was successfully formed with 1 M hexanol. RM formation was confirmed *via* DLS and verified to be monodisperse and small, indicative of RMs (Fig. 2(B)–(D)). MI-RMs are 5.1 ± 1.4 nm, PM-RMs are 6.1 ± 1.4 nm, and ER-RMs are 7.1 ± 2.0 nm, with the standard deviation being that of the distribution of the diameters of the mmRMs (Fig. 2(B)–(D)). Standard errors for both the diameter and standard deviation are reported in Fig. 2. The properties measured by DLS indicate that the RMs are relatively small in size and monodisperse in distribution which allow use in protein NMR and other studies. These results provide new models which can be used to replicate biological systems to study membrane properties, an improvement in biological accuracy compared to more homogenous membrane models.

While DLS provides size and distribution properties, shape is not readily apparent in these measurements. A nearly spherical shape is often advantageous, however, is not strictly required for optimally housing proteins within RMs for NMR study.^53^ To understand the shape of RMs formulated here we used cryo-electron microscopy (cryo-EM) imaging (Fig. 2(E)–(G)). Images captured approximately spherical RMs with a major population with a ∼10 nm size, slightly larger than the apparent size measured by DLS. Size dissimilarities may reflect the differences in the RM components that contribute to the measured solvodynamic radius in DLS *versus* the components that provide contrast in cryo-EM. Additionally, a second minor population of smaller RMs is apparent in the cryo-EM images, which was not observed in the DLS size distributions. It is possible that the cryogenic freezing process may impact the size and may also result in the population of smaller RMs that are observed, which may account for some of the observed differences between DLS and cryo-EM. Nevertheless, cryo-EM imaging clearly indicates roughly spherical RMs, which is consistent with the DPC-DLPC formulation.^35^

**Fig. 3:**
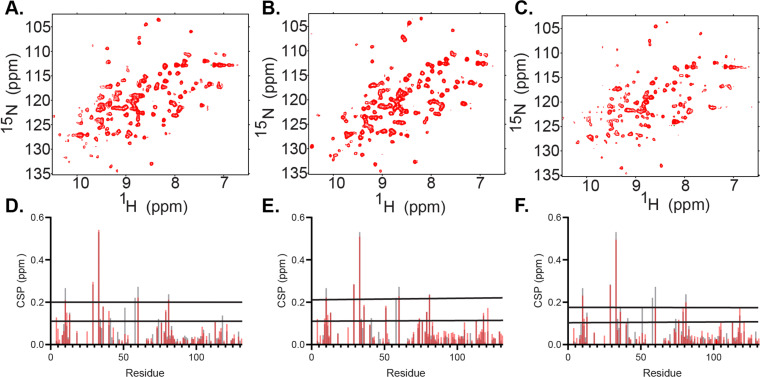
Encapsulation of fatty acid binding protein 4 (FABP4) within the three biologically inspired RM formulations. (A) ^15^N-HSQC of 164 μM FABP4 encapsulated in the ER-RM RM formulation with using 100 mM surfactants and 800 mM of hexanol. (B) ^15^N-HSQC of 164 μM FABP4 encapsulated in the MI-RM formulation using 100 mM surfactants and 900 mM of hexanol. (C) ^15^N-HSQC of 164 μM FABP4 encapsulated in the PM-RM formulation using 100 mM surfactants and 1 M of hexanol. All spectra were collected at 600 MHz at 25 °C using pentane as the solvent. (D) Chemical shift perturbations (CSPs) show membrane interactions and similar shifts for the PM-RM (red) as for the DLPC:DPC RM (gray).^38^ (E) CSPs show membrane interactions and similar shifts for the MI-RM (red) as for the DLPC:DPC RM (gray).^38^ (F) CSPs show membrane interactions and similar shifts for the ER-RM RM (red) as for the DLPC:DPC RM (gray).^38^

FABP4 was used to test the ability of the biologically inspired RM formulations to house membrane associated proteins for NMR study. FABP4 is known to distribute fatty acids from lipid droplets in adipocytes to a variety of cell membranes, with the plasma membrane known as a primary destination for these lipids.^62^ Therefore, the PM-RM formulation represents the most functionally relevant protein-membrane interaction. However, to fully benchmark, all three membrane-specific formulations were used to successfully encapsulate FABP4 (14.6 kDa). Recombinantly expressed and purified FABP4 yielded high-quality, well-dispersed ^15^N-HSQC spectra, suggesting proper fold and function. Previously, the confined interior of DLPC:DPC mmRMs or nRMs was shown to induce direct observation of the membrane-bound state of FABP4, which is otherwise difficult to observe. The protein was added in its lyophilized form after each mmRM was constructed using a *W*_0_ of 20 and 100 mM surfactants. The hexanol concentration necessary for encapsulation of FABP4 within the MI-RM is 900 mM, 800 mM for the PM-RM, and 1 M for the ER-RM. DLS of FABP4 encapsulated in each of the formulations was measured with diameters and standard deviations (the distribution range of the diameters) as follows: MI-RM is 5.7 ± 1.2 nm, PM-RM is 5.6 ± 1.5 nm, ER-RM is 5.1 ± 1.5 nm (Fig. 2(B)–(D)). The RMs retain their small size and uniform distribution upon encapsulation of a protein. These values are all similar to the diameter of the RMs without protein, indicating only small changes to the size upon encapsulation. Confirmation of encapsulation was determined by ^15^N-HSQC spectra of FABP4 in each of the RM formulations, yielding high-quality spectra indicative of a well-folded protein (Fig. 3(A)–(C)).^35,37^ Chemical shift perturbations (CSPs) measure the degree of change in resonance frequency in NMR spectra, such as ^15^N-HSQCs, upon a perturbation of a protein on an approximately per-residue basis. CSP calculations comparing the encapsulated protein to aqueous protein reveal any interactions between protein and RM. Our results confirmed that the residues speculated to be involved in membrane binding in FABP4 showed increased CSPs when FABP4 was encapsulated, in all three bio-inspired formulations (Fig. 3(D)–(F)).^63^ While the overall CSP pattern is similar among the three membrane mimetic formulations, there are minor differences. These may be from slightly different lipid interactions or surface properties due to the variation in lipid content for each RM formulation. CSPs in the three bio-inspired RMs corresponded well to CSPs for FABP4 encapsulated in DLPC:DPC mmRMs and nRMs, confirming that the expected interaction between FABP4 and the membrane is captured.^35,37^ We note here that direct observation of the membrane interface of FABP4 is challenging due to its weak and transient nature with other membrane models, but is captured in RMs due to confinement within a membrane model.^64^ CSP mapping indicates shifting greater than the 20% trimmed mean plus 1σ for the ER-RM includes F4, K9-V11, S13, T29, A33, A36, I51, T60, K79, K81, and L113 (Fig. 3(D)). CSP shifting for the MI-RM includes the same residues as the ER-RM except A36 and includes T74, D76, V118, and G121 (Fig. 3(E)). The CSP shifting over the 20% trimmed mean plus 1σ for the PM-RM includes L10-V11, S13, T29, A33, A36, M40, T60, D76, K79, K81, L113, V118, and G121 (Fig. 3(F)). This shifting is expected based on the proposed region of membrane interaction which includes the helices comprising of residues F16-L23 and F27-V32.^65^ Non-membrane interacting residues may be used as markers for observing any potential pH effects upon encapsulation.^37^ There were small CSPs in non-membrane interacting, pH-sensitive residues, that correspond well to a pH of around 6.5 for the RM interiors, as expected.^37^

**Fig. 4:**
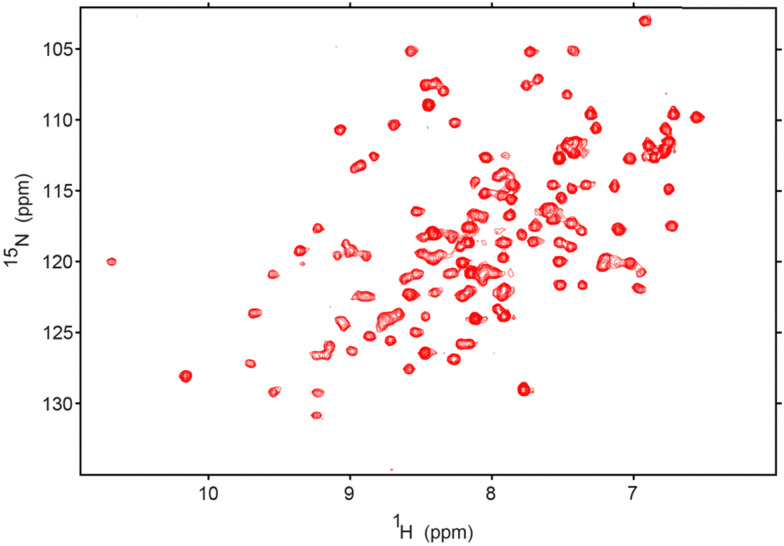
^15^N-HSQC of 120 μM SCP2, including *E. coli* endogenous lipids, encapsulated in 100 mM total lipids of the PM-RM formulation at pH 8.5. This demonstrates the initial successful encapsulation of SCP2 in the PM-RM in its native folded state. The *W*_0_ is 20 with 800 mM hexanol, the NMR spectrum was collected on a 600 MHz NMR at 25 °C.

**Fig. 5:**
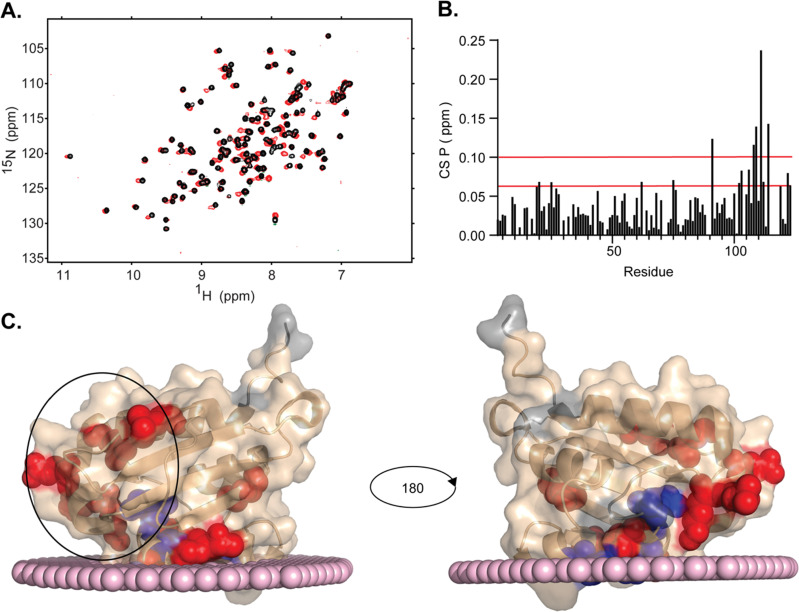
Encapsulation of SCP2 in PM-RM allows mapping of the membrane interface. (A) ^15^N-HSQC of 4:1 sodium cholate hydrate with delipidated aqueous 120 μM SCP2 (black) and delipidated 120 μM SCP2 with 5 mM sodium cholate hydrate (red) in 100 mM PM-RM with a *W*_0_ = 20 and 800 mM hexanol as cosurfactant and hexane as the solvent. Data was collected on a 600 MHz NMR at 37 °C. (B) CSPs between aqueous SCP2 with sodium cholate hydrate and SCP2 with sodium cholate hydrate in a PM-RM. Red lines represent the 20% trimmed mean plus 1σ (0.06 ppm) and 2σ (0.10 ppm). (C) PPM 3.0 server^72^ calculation of SCP2 (pdb code: 1C44) interacting with a model of mammalian plasma membrane (surface depicted with light pink spheres). NMR results are mapped onto the protein structure, represented by the wheat cartoon with unassigned peaks represented in gray and the spheres representing the residues with significant CSPs (red: 20% trimmed mean plus 1σ; blue: 20% trimmed mean plus 2σ). Black circle highlights the residues in and adjacent to a potential secondary region previously suggested to interact with the membrane.^73^

To extend the utility of biologically inspired RM formulations for observing difficult to capture LTP–membrane interactions, we pursued mapping of the membrane interaction surface of SCP2. SCP2 (13.3 kDa) is an important lipid transport protein with roles in various cellular processes, including metabolism of sterols and other lipids and their transport among membranes.^39–43^ This protein, until now, has not been studied at high resolution in its membrane-bound state. The PM-RM was chosen as the membrane model due to the tendency of SCP2 to interact with the PM, often to transport cholesterol.^39^ The PM-RM also most closely resembles the peroxisome, where SCP2 is known to localize, whereas the MI-RM and ER-RM have lipid contents which are not consistent with the peroxisome.^66^ However, to further test compatibility of MI-RM and ER-RM formulations we tested these systems and found that SCP2 encapsulates well within both models (ESI,† Fig. S3). Initial trials show successful encapsulation of SCP2 with better signal and resolution at pH 8.5 *versus* lower pH values. SCP2 at pH 8.5 was successfully encapsulated in a 100 mM PM-RM and confirmed *via*^15^N-HSQC NMR (Fig. 4), resulting in a high-quality spectrum, motivating us to further investigate membrane interactions. High-quality, well-dispersed signals in the ^15^N-HSQC indicate a properly folded protein. However, further scrutiny revealed that the standard purification of SCP2 without further processing, in our hands, results in protein that is bound to endogenous lipid, which is common for lipid carrier proteins.^67^ This was confirmed upon the successful delipidation of SCP2 using Lipidex-5000, confirmed *via*^15^N-HSQC, comparing holo and delipidated SCP2 (ESI,† Fig. S4A).^68^ Complete delipidation was also confirmed by the addition of sodium cholate hydrate, a known bile salt ligand,^42^ to the delipidated SCP2 which showed reversion to ^15^N HSQC spectrum that is very similar to the protein before delipidation (ESI,† Fig. S4B), further indicating that SCP2 was in its holo-form before delipidation. The cholate binding results also confirm that the recombinantly expressed and purified SCP2 is functionally competent and able to bind to a known ligand. We decided to continue experimentation with delipidated SCP2 to have better control over the ligand rather than use samples containing the likely heterogenous copurified *E. coli* lipids. Initial encapsulation trials of apo-SCP2 in the DLPC:DPC system results in large CSPs in the residues that cover nearly the entire protein, indicating a spectral effect that is likely from both membrane interactions and lipid binding within the cavity (ESI,† Fig. S5). We elected to use a ligand bound form to encapsulate SCP2 for further study to avoid mistaking spectral changes upon lipid binding for membrane interactions.^41^ Delipidated SCP2 with 5 mM sodium cholate hydrate was optimized and successfully encapsulated in a 75 mM 50 : 50 molar ratio DLPC:DPC mmRM (ESI,† Fig. S6). The optimized mmRM was solubilized in hexane and ^15^N-HSQC data was collected at 37 °C with a buffer pH of 6.0. The high-quality spectrum indicates a well-folded protein is encapsulated under these conditions. These conditions were then transferred to the PM-RM formulation, where a spectrum of SCP2 in its membrane bound form was collected (Fig. 5(A)). Successful collection of the ^15^N-HSQC of SCP2 with sodium cholate hydrate in the PM-RM allowed comparison with the aqueous form bound to sodium cholate hydrate (Fig. 5(A)). Residue assignments were collected through a series of 3D NMR experiments including HNCO, CBCACONH, and HNCACB at both pH 7.4 and pH 6.0 (37 °C) on aqueous protein to confirm the previously published assignments.^43^ The RM residue assignments were transferred from the aqueous assignments. 94% of the residues (excluding prolines) were successfully assigned. Assignments were made for the following residues: S3-G6, A9-G42, G44-L62, N64-N89, Q91-L114, and G119-L123. The pH of the interior of RMs may be driven by titratable headgroups, as observed previously with nRMs, and may cause peak shifting in protein NMR spectra. An aqueous pH titration of SCP2 from pH 6.0 to 8.5 was completed and there was no noticeable peak shifting from the lipid headgroups, indicating that a potential pH change would not greatly impact peak positions and would not interfere with CSP mapping (ESI,† Fig. S7).^37,69^ The CSP comparison indicates a membrane interaction site of SCP2, separate from the sodium cholate hydrate ligand binding pocket effects (Fig. 5(B)). SCP2 has several shifting resonances with CSPs greater than the 20% trimmed mean plus 1σ (0.06 ppm) including L20, E25, L62, M75, T102-G103, G106, Q112, and K122-L123. The CSPs also indicate several resonances with greater shifting than the 20% trimmed mean plus 2σ (0.10 ppm) including Q91, A108-M109, L111, and L114. Mapping these on the crystal structure of SCP2 (pdb code: 1C44),^70^ there are clear indications of regions affected by the encapsulation. The region towards the C-terminus (∼20 residues) with high CSPs is consistent with the structure interacting with a mammalian plasma membrane determined by the PPM 3.0 server – a computational method for determining energetically optimized positions of proteins against membranes (Fig. 5(C)).^71^ The PPM calculation allows a prediction of the structure of SCP2 in its membrane bound state from the aqueous crystal structure, which matches well, however not perfectly, with the highest shifting resonances (Fig. 5(C)). Through mapping of the CSP residues onto the crystal structure, we observe an additional region that has been implicated in membrane interactions through observation of binding of peptide derived from the N-terminus of the SCP2 sequence.^67,72^ The residues displaying significant shifting are residues L20 and E25 within the N-terminal region and the adjacent K122-L123 (Fig. 5(C)). The CSPs of this region may be a secondary membrane interacting site as alluded to by a previous N-terminal peptide interaction study.^72^ The region displaying CSPs overlays well with a lysine rich region (K14, K18-K19, K29-K30) suggesting that these cationic residues may drive the interaction. By encapsulating SCP2 within the PM-RM, we have directly observed the protein in its membrane bound state for the first time at high-resolution, in a membrane model that is more biologically relevant than standard models. By encapsulating proteins within an RM system which reflects the membranes where they may be naturally found, new areas of study may be opened where the function, structure, and dynamics of these proteins may be studied *in vitro* with conditions that better resemble biological membranes.

## Conclusions

The goal of this study was to advance formulations of mmRMs towards the lipid content found in specific biological membranes. We have tested the ability of multiple lipids to incorporate into mmRMs and found that the DLPC:DPC system tolerates a wide range of lipid types up to and beyond percentages that are found in selected biological membranes. The breadth of lipids incorporated in this study only reflects variation in the headgroup chemistry; exploration of RM tolerance to lipid tail chemistry will be explored in future work. Size and shape characterization indicate small and monodisperse RMs upon incorporation of all tested lipids, which are necessary for protein NMR and other biophysical applications. These results suggested that more biologically accurate membrane models may be formulated. Subsequently, we tested three biologically inspired mmRM formulations that reflect specific membrane leaflets in eukaryotic cells: inner leaflet of the plasma membrane, outer leaflet of the outer mitochondrial membrane, and outer leaflet of the rough endoplasmic reticulum membrane. These formulations proved to be small and monodisperse and successfully encapsulated FABP4 and SCP2, which are membrane interacting lipid carrier proteins. Finally, we have applied the plasma membrane RM formulation to map the first high-resolution membrane interaction with SCP2. NMR spectral analysis highlights the membrane interacting site as well as a potential secondary interacting site based on CSPs, correlating with both the predicted membrane interactions and with previous investigation of the N-terminal peptide binding to membrane models.^72^ Utilizing mmRM formulations that reflect cellular membranes allows a higher degree of confidence in studying biologically relevant protein–membrane interactions.

The biologically inspired RMs presented here have advantages and drawbacks compared to other membrane models that should be considered when designing experimental approaches. The curvature of the interior of the RMs is extreme compared to the vast majority of biological membranes, making bicelles and nanodiscs better models of planar biological membrane surfaces. However, the interface of small proteins, such as those demonstrated here, likely have only a small curvature deviation overall from the interface of a more planar membrane surface. While mmRMs are known to greatly extend the sample lifetimes of proteins that may have limited stability in other models such as micelles, we have found that a small percentage of proteins are incompatible, likely due to sensitivity from exposure to alkanes.^25,35^ While liposomes may be constructed from complex mixtures of lipids, their large size excludes them from use in protein NMR applications.^73^ Similarly, isotropic bicelles and nanodiscs have been constructed from complex lipid mixtures from native sources, however they often necessitate perdueteration of the hosted proteins and the lipids or surfactants to achieve ample protein NMR spectral quality.^24,74^ Micelles, while among the smallest membrane models and best performing for NMR experiments, are typically constructed from artificial surfactants that do not well-reflect the chemistry of lipids found in cellular membranes. An advantage of using RMs in protein NMR applications is the low-viscosity solvent that results in relatively fast rotational diffusion, allowing for narrow line shapes despite the somewhat large size of the RM particle.^29^ The confined interior of mmRMs provides a unique environment for enhancing weak-binding events, such as low-affinity small-molecule ligands, as demonstrated previously,^33,34^ and transient protein–membrane interactions as demonstrated here. Finally, the versatility of the mmRM system allows for incorporation of a large range of lipids allows for custom formulations that may reflect biological membranes of interest or other experimentally necessary parameters. This represents a powerful addition to the set of tools available for studying membranes and their associated proteins.

## Author contributions

Sara H. Walters: methodology; visualization; writing – original draft; writing – review & editing; investigation. Rachel L. Signorelli: data curation: investigation; writing – review & editing. Allyson G. Payne: data curation; investigation; writing – review & editing. Alimohammad Hojjatian: methodology; data curation; writing – review & editing. Brain Fuglestad: conceptualization; funding acquisition; data curation; project administration; supervision; writing – review & editing.

## Data availability

The data supporting this article have been included as part of the ESI.†

## Conflicts of interest

There are no conflicts to declare.

## Supplementary Material

SM-021-D5SM00033E-s001
