# Supplementary material for: Compositional versatility enables biologically inspired reverse micelles for study of protein–membrane interactions
Source: Soft Matter. 2025 Apr 4;21(18):3547–57. doi: 10.1039/d5sm00033e (PMC11984498; doi:10.1039/d5sm00033e)
Supplement: SM-021-D5SM00033E-s001 [file SM-021-D5SM00033E-s001.pdf]

Supplementary Information (SI) for Soft Matter.
This journal is © The Royal Society of Chemistry 2025

Supporting Information for:

Compositional versatility enables biologically inspired reverse micelles for study of protein-membrane interactions

Sara H. Walters[1], Rachel L. Signorelli[1], Allyson G. Payne[1], Alimohammad Hojjatian[2], Brian Fuglestad[1,3,*]

[1] Department of Chemistry, Virginia Commonwealth University, Richmond, Virginia, USA

[2] Office of the Vice President for Research and Innovation, Virginia Commonwealth University, Richmond, VA, 23298, USA

[3] The Center for Drug Discovery, Virginia Commonwealth University, Richmond, VA 23298, USA.

*Correspondence: fuglestadb@vcu.edu

Protein Expression

The gene encoding human FABP4 with a poly-histidine tag and human SCP2 with a poly-histidine tag were synthesized by Genescript and subcloned into pET-28a(+) vectors. Each plasmid was transformed into BL21 (DE3) *E. coli,* which were grown on LB-agar plates with kanamycin. Glycerol stocks were prepared from single colonies on the agar plates and used to seed overnight cultures in M9 minimal media at 37°C. The cultures were then pelleted and used for growth in 1L M9 minimal media with 1 g/L $^{15}NH_4Cl$ and 2 g/L ^{13}C-D-glucose, if needed, for isotopically labeling the proteins. The cells were grown until the OD_{600} measured 0.8. The cells were then induced with 1 mM isopropyl-β-D-1-thiogalactopyranoside (IPTG) overnight at 18°C for FABP4 and 30°C for SCP2. Cells were harvested the next day by centrifugation.

Protein Purification

FABP4 was purified based on previously published protocols.[1,2] FABP4 was purified by resuspending and lysing the cells in lysis buffer [100 mm Tris pH 7.4, 500 mM NaCl, 0.5% Triton v/v, 0.1 mg/mL lysozyme, protease inhibitor cocktail, and 2 mM dithiothreitol (DTT)]. The cells were then sonicated. The FABP4 was purified via Ni-NTA affinity chromatography and the His-tag was cleaved using TEV protease in an overnight dialysis [20 mM Tris pH 7.4, 100 mM NaCl, and 2 mM DTT]. The protein was repurified the following day and again dialyzed overnight into NMR buffer [20 mM Tris pH 7.4, 100 mM NaCl, and 2 mM DTT]. A ^{15}N-HSQC NMR spectrum of FABP4 was collected to confirm the presence of bound, endogenous lipids from the *E. coli,* bound to saturation.

SCP2 purification was based on a previously published protocol.[3] SCP2 purification began with resuspension and lysis of the harvested cells in lysis buffer [100 mM Tris pH 7.4, 500 mM NaCl, 0.5% v/v Triton, protease inhibitor, 0.1 mg/mL lysozyme, and 2 mM DTT]. The cells were sonicated separated using a Ni-NTA affinity column. The His-tag was cleaved using TEV protease in an overnight dialysis [20 mM Tris pH 7.4, 100 mM NaCl, 5 mM imidazole, and 2 mM DTT]. The protein was repurified the following day and dialyzed into NMR buffer [20 mM Tris or Bis-Tris pH 6.0, 7.4, or 8.5, 100 mM NaCl, and 2 mM DTT] overnight.

SCP2 delipidation was based on a previously published protocol for delipidation of FABP4.[4] 1 g of Hydroxyalkoxypropyl-Dextran (Lipidex-5000 resin) per liter of SCP2 preparation is equilibrated at 37°C for 2.5 hours with 1 mL of 10 mM potassium phosphate pH 7.4, 150 mM potassium chloride, and 0.2 g/L sodium azide. SCP2 protein from the repurification process is added to the resin and incubated at 37°C for an additional 2 hours. The protein is then collected by filtering out the resin and was subsequently dialyzed overnight into NMR buffer at pH 6.0.

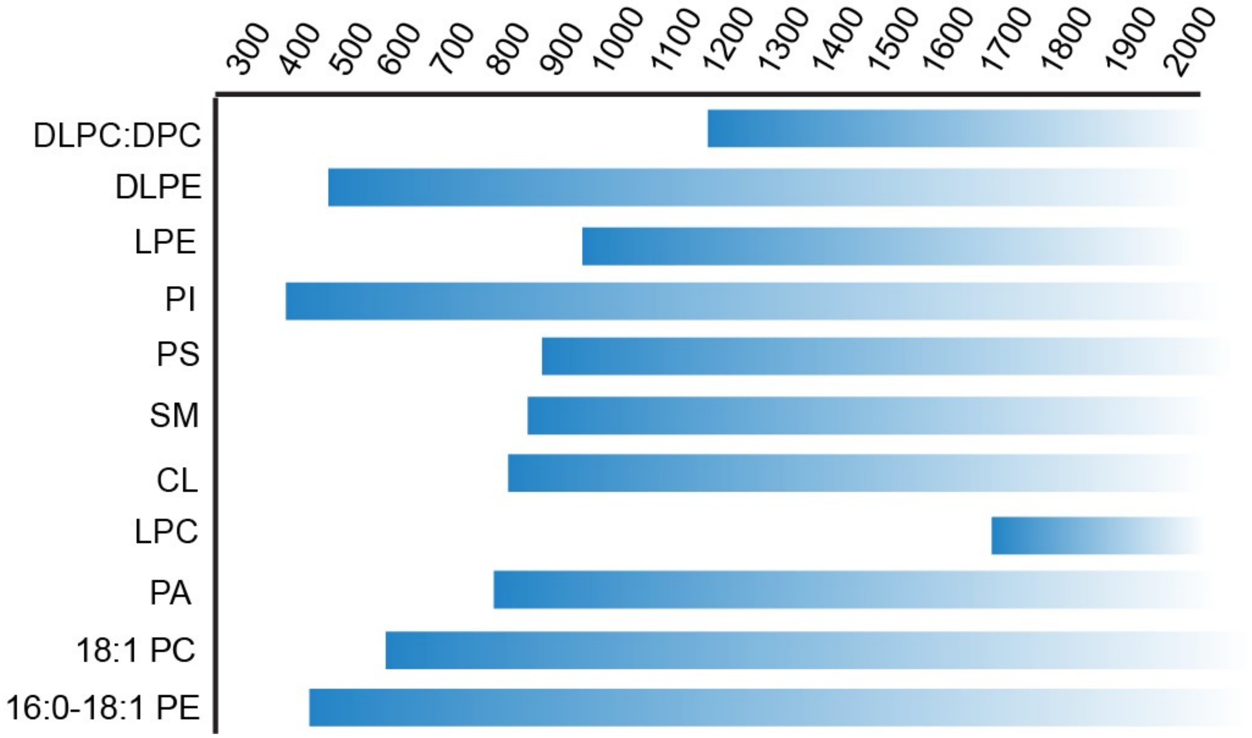

Supplementary Figure 1. Hexanol phase diagrams for the formation of the reverse micelles with the maximum tolerance for each surfactant. Where the blue bar begins, the reverse micelle showed visual clarity, which remained through a hexanol titration of up to 2 M for all systems. The lipid of interest for each RM tolerance test is listed, see **Figure 1B** for compositions.

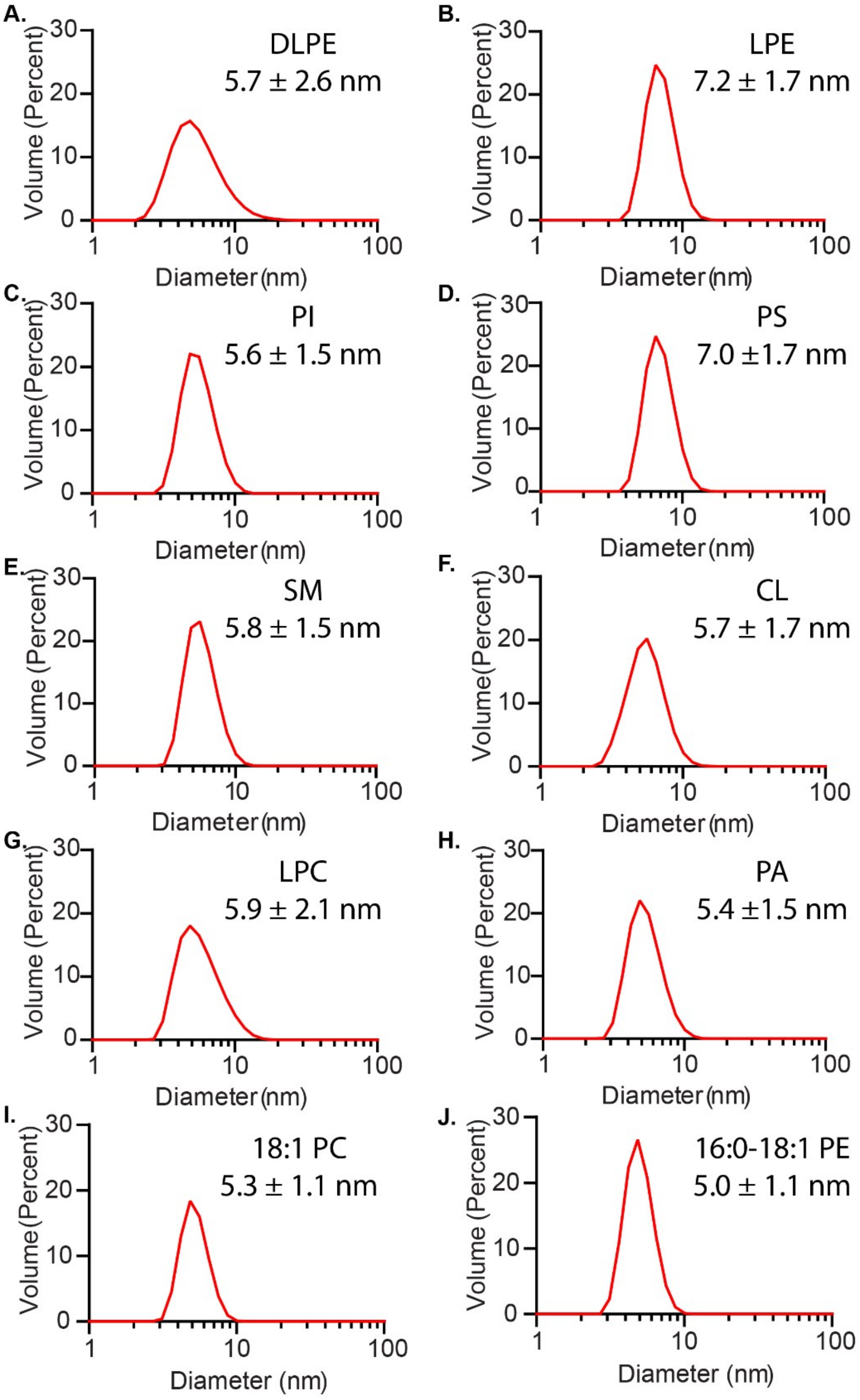

Supplementary Figure 2. DLS confirmation of monodisperse, approximately spherical, and small reverse micelles during tolerance testing. Fitted diameter and standard deviation, reflecting the distribution width, are included here. See **Figure 1B** for formulation details and standard error values.

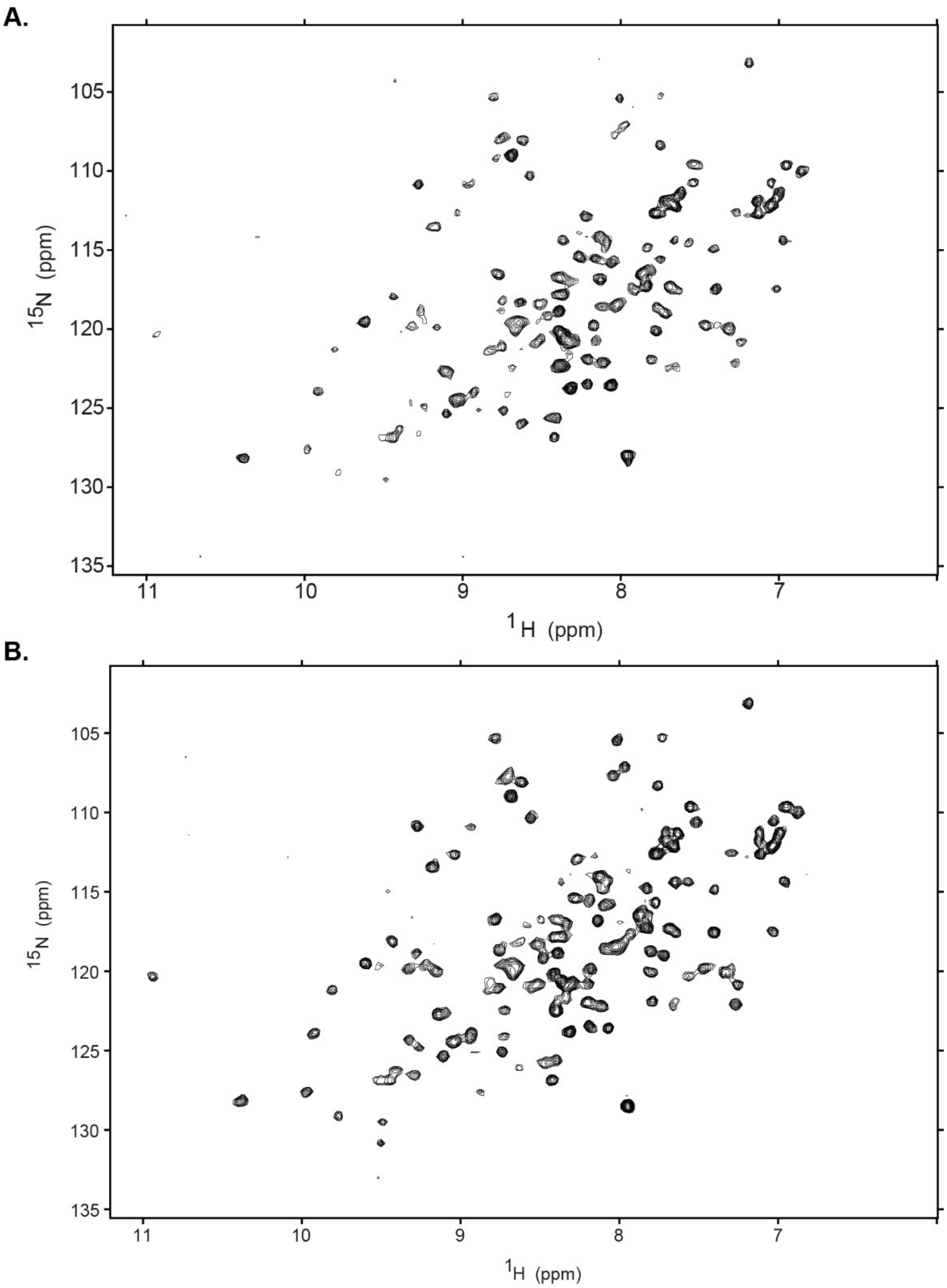

Supplementary Figure 3. Encapsulation of apo-SCP2 in mmRMs. **A.** ^{15}N-HSQC of encapsulated, delipidated 120 μM SCP2 with 5 mM sodium cholate hydrate in a 100 mM ER-RM. The RM had a W_0 of 20 with 1 M of hexanol. **B.** ^{15}N-HSQC of encapsulated, delipidated 120 μM SCP2 with 5 mM sodium cholate hydrate in a 100 mM MI-RM. The RM had a W_0 of 20 with 1 M of hexanol. Both NMR experiments were collected on a 600 MHz NMR at 37°C and at pH 6.0.

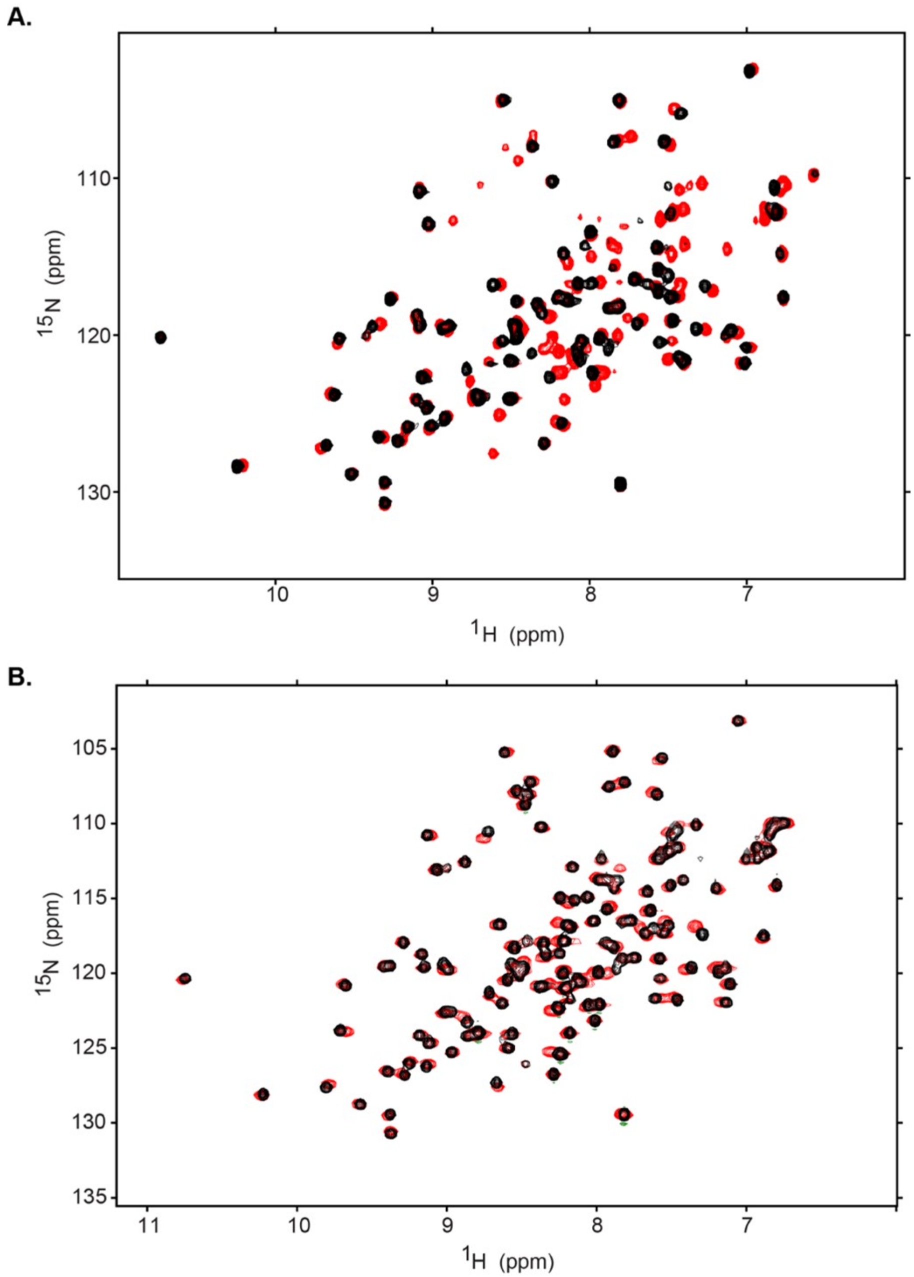

Supplementary Figure 4. Spectral demonstration of the presence of endogenous lipids and delipidation of SCP2. **A.** ^{15}N-HSQC showing the successful dilapidation of 120 µM SCP2 (black) compared to the 120 µM SCP2 copurified with *E. coli* endogenous lipid (red) at pH 8.5. **B.** ^{15}N-HSQC confirming the addition of 4:1 sodium cholate hydrate to 120 µM SCP2 (black) correlates to the 120 µM SCP2 with endogenous lipids (red) at pH 6.0 and 37°C.

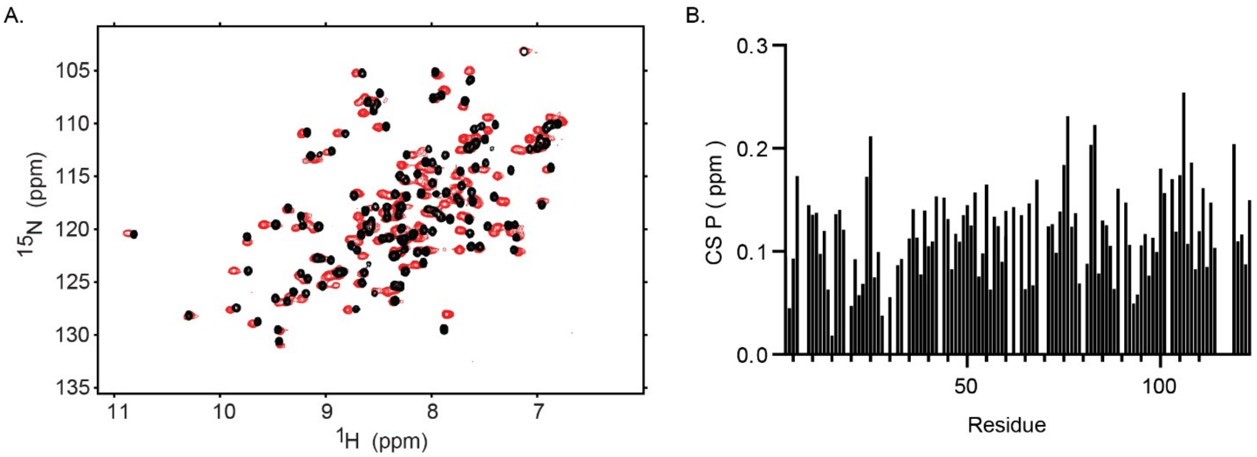

Supplementary Figure 5. Encapsulation of apo-SCP2 in mmRMs. **A.** ^{15}N-HSQC comparing the delipidated aqueous 120 µM SCP2 to the encapsulated, delipidated 120 µM SCP2 in a 75 mM 50:50 molar ratio of DLPC:DPC RM. The RM had a W_0 of 20 with 900 mM of hexanol. Both NMR experiments were collected on a 600 MHz NMR at 37°C and at pH 6.0. **B.** CSPs between delipidated SCP2 and encapsulated, delipidated SCP2 showing changes throughout the protein concluding that the protein is further affected by encapsulation than just the membrane interacting site, likely from lipid binding to the central cavity.

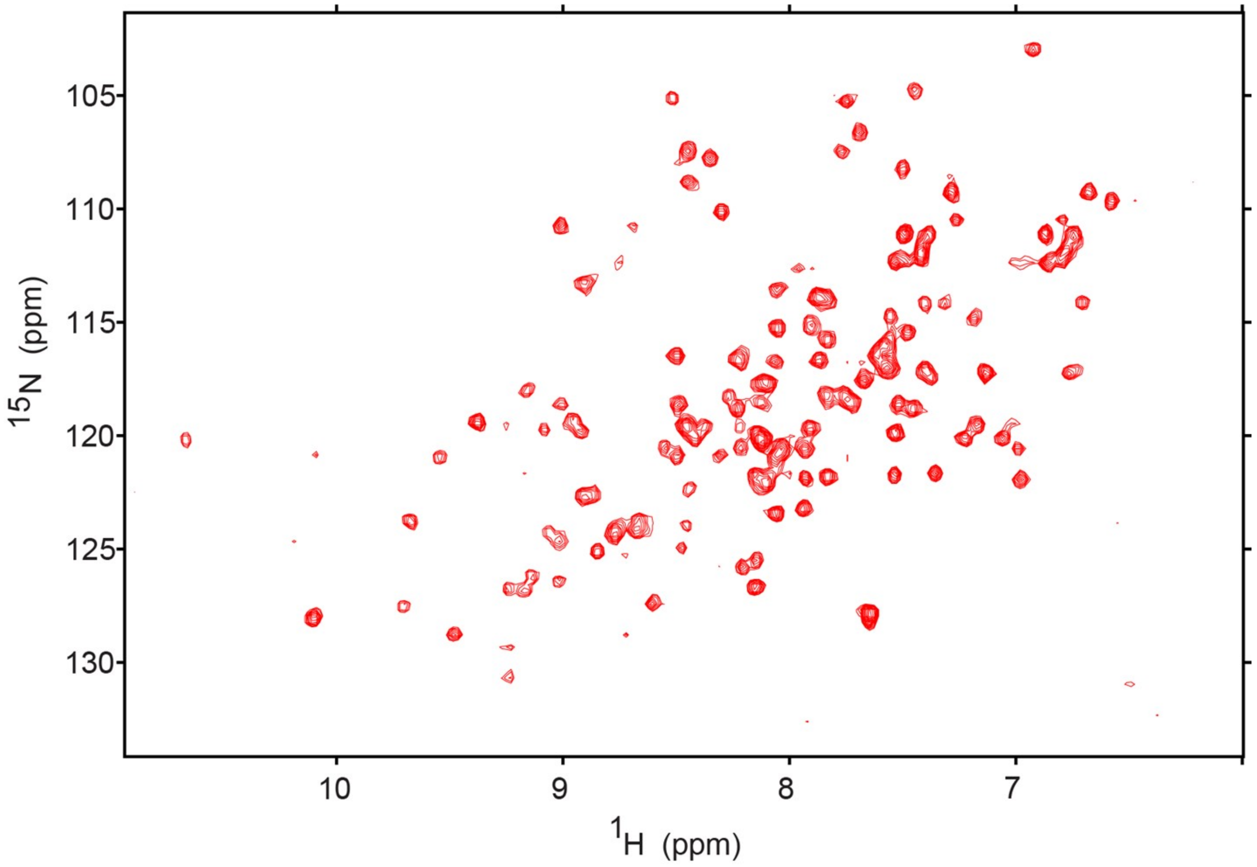

Supplementary Figure 6. ^{15}N-HSQC shows successful encapsulation of 120 µM delipidated SCP2. Protein was encapsulated at pH 6.0 in 75 mM 50:50 molar ratio DLPC-DPC with 5 mM sodium cholate hydrate. W_0 is 20 with 1.4 M hexanol added as a cosurfactant. Data was collected at 37°C in hexane with a 600 MHz NMR.

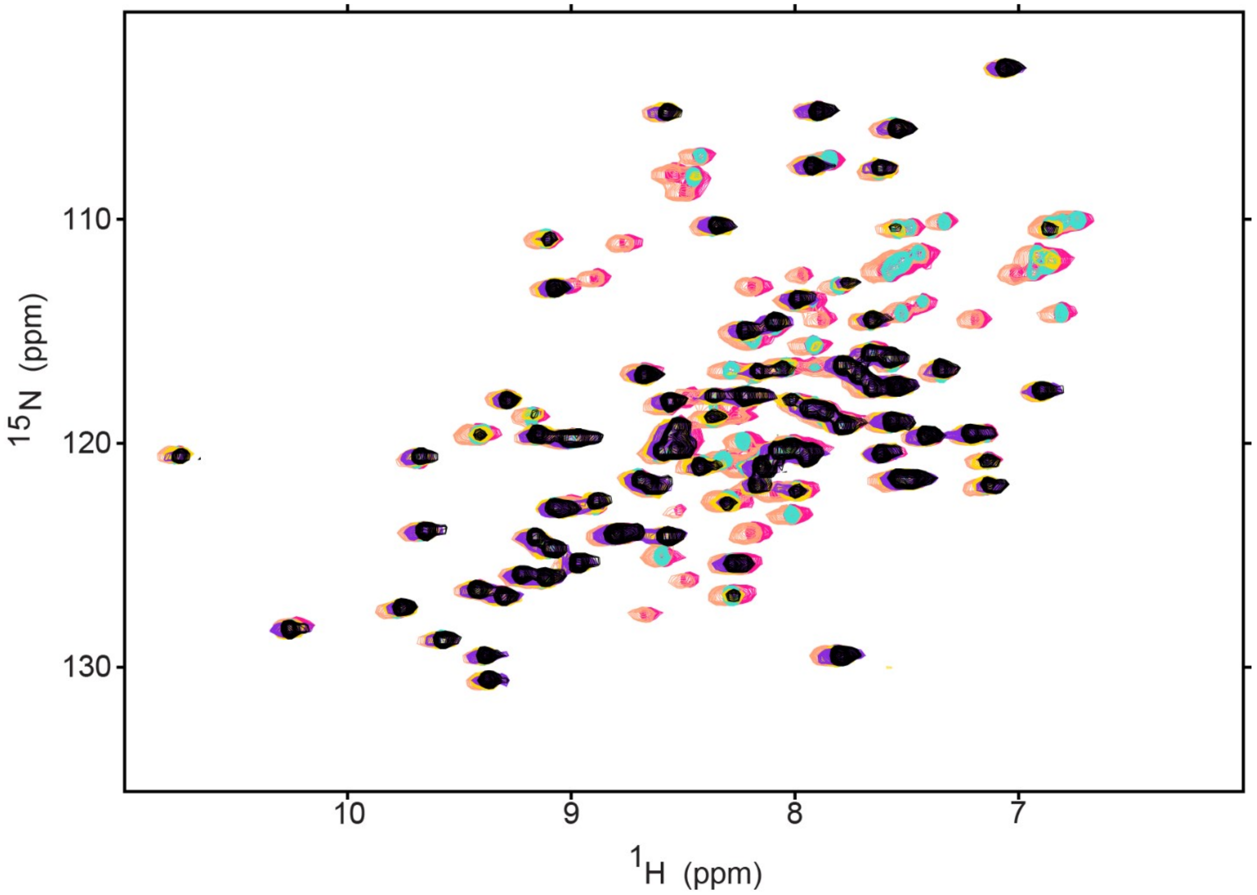

Supplementary Figure 7. ^{15}N-HSQC pH titration of delipidated aqueous SCP2 from pH 6.0 to 8.5. Spectra were collected at 37°C on a 600 MHz NMR. The pH titration shows limiting shifting of peaks due to pH change and instead shows line broadening due to the pH increase. pH 6.0: pink; pH 6.5: salmon; pH 7.0: turquoise; pH 7.5: gold; pH 8.0: violet; pH 8.5: black.

Supplementary References

1 S. H. Walters, A. J. Castillo, A. M. Develin, C. L. Labrecque, Y. Qu and B. Fuglestad, *Protein Science*, DOI:10.1002/pro.4786.

2 C. L. Labrecque, A. L. Nolan, A. M. Develin, A. J. Castillo, A. R. Offenbacher and B. Fuglestad, *Langmuir*, 2022, **38**, 3676–3686.

3 D. J. Manfra, C. L. Baum, E. Reschley, D. Lundell, P. Zavodny and B. Dalie, *Protein Expr Purif*, 1995, **6**, 196–205.

4 Q. Wang, S. Rizk, C. Bernard, M. P. Lai, D. Kam, J. Storch and R. E. Stark, *Biochem Biophys Rep*, 2017, **10**, 318–324.
